# A Rare Case of Hepatic Abscess That Resolved after Drainage of Pleural Empyema

**DOI:** 10.3390/healthcare9121732

**Published:** 2021-12-14

**Authors:** Oluwafemi Augustine Ajibola, Taiwo Opeyemi Aremu, Oluwatosin Esther Oluwole, Olawunmi Olayiwola, Nida Khokhar, Matthew Apedo, Valerie Cluzet

**Affiliations:** 1 Department of Medicine, Vassar Brothers Medical Center, Nuvance Health, 45 Reade Place, Poughkeepsie, NY 12601, USA; nida.khokhar@nuvancehealth.org (N.K.); matthew.apedo@nuvancehealth.org (M.A.); valerie.cluzet@nuvancehealth.org (V.C.); 2 Department of Pharmaceutical Care & Health Systems (PCHS), College of Pharmacy, University of Minnesota, 308 Harvard Street SE, Minneapolis, MN 55455, USA; aremu006@umn.edu; 3 Division of Environmental Health Sciences, School of Public Health, University of Minnesota, 420 Delaware Street SE, Minneapolis, MN 55455, USA; 4 Division of Epidemiology & Community Health, School of Public Health, University of Minnesota, 300 West Bank Office Building, 1300 S. 2nd Street, Minneapolis, MN 55454, USA; oluwo006@umn.edu; 5 Department of Medicine, Milton Keynes University Hospital, Standing Way, Eaglestone, Milton Keynes MK6 5LD, UK; olawunmi.olayiwola@mkuh.nhs.uk

**Keywords:** empyema, hepatic abscess, pleural effusion, transdiaphragmatic extension, disseminated pyogenic infection

## Abstract

Empyema has rarely been associated with hepatic abscess. In patients with concurrent empyema and hepatic abscess, hepatic abscess drainage is usually required after drainage of the pleura. We present a rare case of a 91-year-old Caucasian man who presented with a 2-week history of productive cough, fever, shortness of breath, and generalized malaise. The patient was found to have concurrent streptococci empyema and hepatic abscess, and, interestingly, the hepatic abscess resolved after the drainage of the empyema and initiation of antibiotics.

## 1. Introduction

Anginosus group streptococci are part of normal human flora and are widely known for disseminated pyogenic infection. Concurrent empyema and liver abscess have rarely been reported in the literature [1]. Typically, drainage of both the empyema and the liver abscess is required for complete resolution of the hepatic abscess [1,2,3]. We present a rare case of a hepatic abscess that rapidly resolved after drainage of the pleural *Streptococcus anginosus* empyema in an elderly male patient.

## 2. Case Presentation

The patient is a 91-year-old Caucasian man with a past medical history of coronary artery disease, congestive heart failure, atrial fibrillation, hypertension, interstitial lung disease, and obstructive sleep apnea presented with a 2-week history of productive cough, fever, shortness of breath and generalized malaise. On presentation, vitals showed blood pressure of 77/35 mmHg, heart rate of 122 bpm, respiratory rate of 38 bpm, a temperature of 102 F, and oxygen saturation of 98% on 15 L of oxygen. The patient was diaphoretic, with decreased breath sounds in the right lung field, and on palpation of the abdomen, there was right upper quadrant fullness.

**Figure 1:**
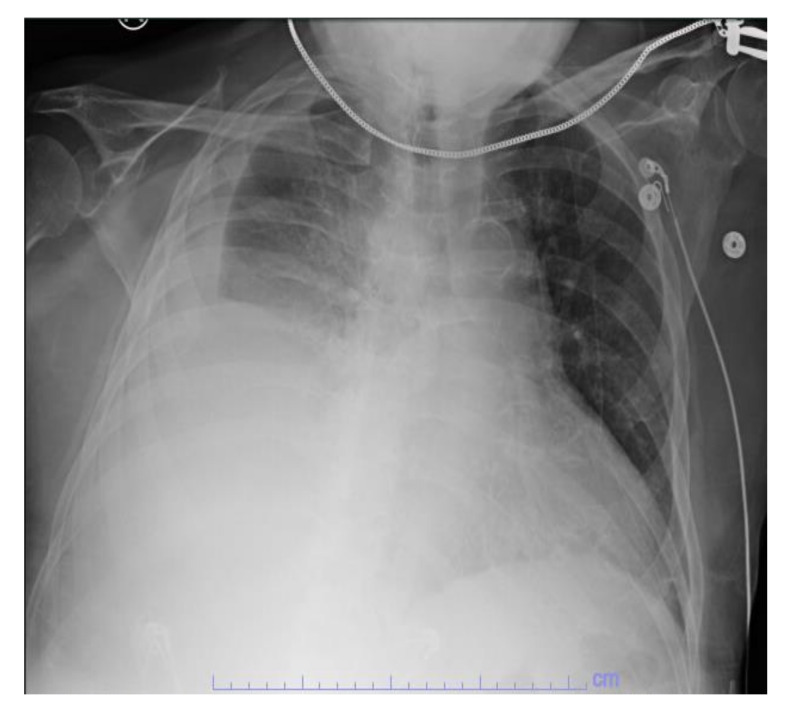
CXR showing acute right pleural effusion.

Initial laboratory studies showed elevated white blood cells (WBC) 22.6 × 10^9^/L with neutrophilia, bicarbonate 21 mmol/L, lactic acid 6.5 mmol/L, anion gap 17, ALT 71 IU/L, AST 69 IU/L, and ALP 450 IU/L. ECG showed atrial fibrillation with a rapid ventricular response. CXR showed acute right pleural effusion (Figure 1). The patient was intubated for respiratory failure. He was also started on antibiotics (piperacillin-tazobactam and azithromycin) and intravenous normal saline with no improvement in blood pressure. The patient was then started on intravenous vasopressor support with norepinephrine and vasopressin and admitted to the intensive care unit (ICU).

**Figure 2:**
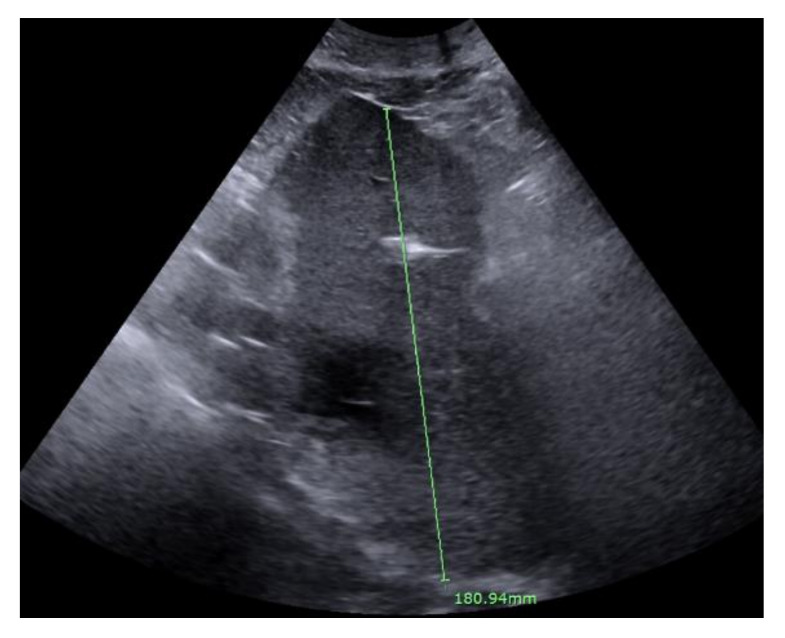
Abdominal ultrasound showing mass-like lesion in the left hepatic lobe.

Due to the right upper quadrant fullness, elevated liver enzymes and fever, an abdominal ultrasound was performed, which showed an acute complex heterogeneous hypoechoic 8 × 7 × 6 cm mass-like lesion in the right hepatic lobe (Figure 2).

**Figure 3:**
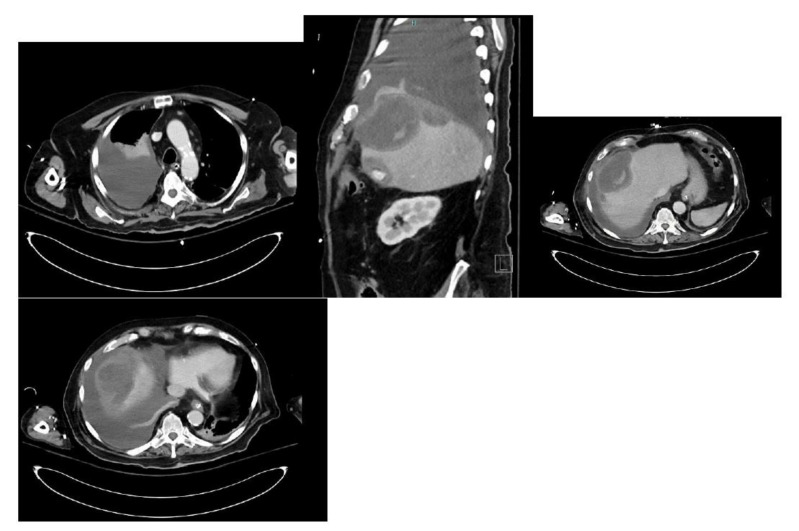
CT abdomen showing a complex low-density right hepatic lobe subcapsular lesion and a moderate right pleural effusion.

To better characterize the lesion, a CT abdomen was done. The CT showed a complex low-density right hepatic lobe subcapsular lesion measuring 13 × 8 × 7 cm, directly abutting the right anterior diaphragm, along with diffuse gross gallbladder wall thickening with cholelithiasis and a moderate right pleural effusion (Figure 3).

**Figure 4:**
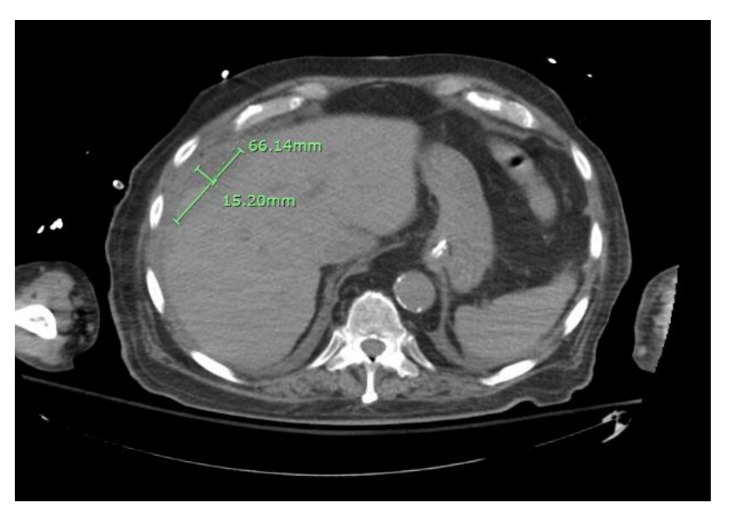
CT abdomen showing reduced size of the right sub-diaphragmatic perihepatic collection.

The patient underwent chest tube placement with the removal of 1600 cc of cloudy light-brown-colored fluid. Pleural fluid analysis was consistent with empyema, with WBC of 70,800 cells/mcL (61% neutrophils), glucose less than 10 mg/dL, LDH of 4821 IU/L, pH of 7.0, protein of 3.6 gm/dL, and Gram stain showing Gram-positive cocci in chains. Cytology was negative for malignant cells but showed severe acute inflammation and rare mesothelial cells. The blood culture on admission grew beta-hemolytic streptococci. The pleural fluid culture grew *Streptococcus anginosus*. On day two of hospitalization, the patient was scheduled for CT percutaneous drainage of liver abscess. However, the CT revealed a significant decrease in the size of the right subdiaphragmatic perihepatic collection to 1.5 cm in greatest thickness (Figure 4).

**Figure 5:**
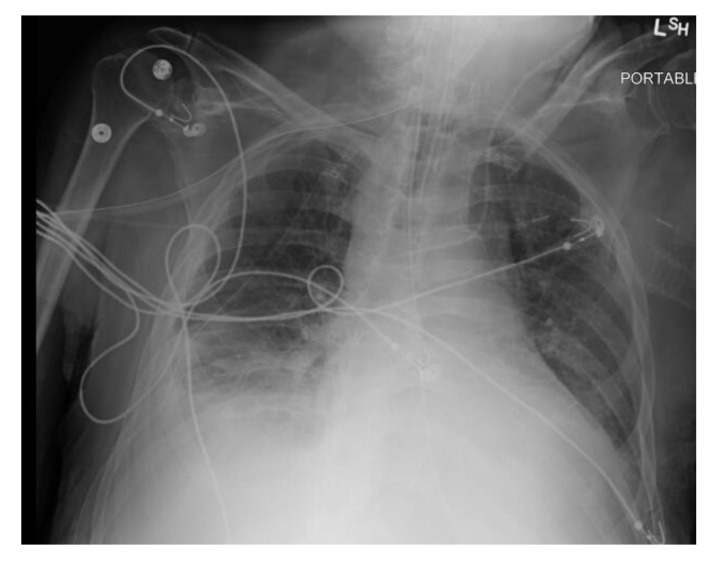
CXR after drainage of pleural empyema.

Due to the improvement of the hepatic collection, the CT-guided drainage of the collection was not performed. Following the persistence of pleural effusion and decreased effluent from the chest tube, an intrapleural thrombolytic was instilled via chest tube with the removal of 1.5 L exudative fluid. Repeat CXR showed improvement in the right pleural effusion (Figure 5), and the patient was successfully extubated on day three of hospitalization.

**Figure 6:**
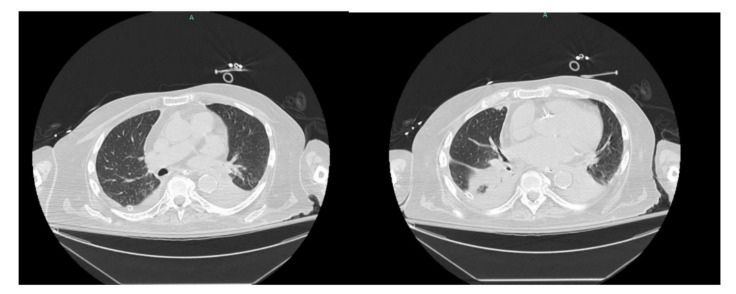
CT chest showing diffuse narrowing of the left mainstem bronchus with trace right pleural effusion.

His hospital course was subsequently complicated by new onset and worsening respiratory distress on day eight of hospitalization requiring re-intubation. Repeat CT chest showed diffuse narrowing of the left mainstem bronchus, occlusion of right posterior segmental bronchi with atelectasis, complete collapse of the left lower lobe due to occlusion of left lower lobe central bronchi with trace right pleural effusion (Figure 6).

Emergent flexible fiberoptic bronchoscopy was done and showed copious thick mucopurulent secretions that were suctioned to clear. The bronchoalveolar lavage grew *Candida albicans*, which was not thought to be a pathogen. After two weeks of hospitalization and the inability to wean the patient from the ventilator, the patient was terminally extubated, and care was focused on comfort.

## 3. Discussion

Empyema is a collection of fluid containing immune cells, dead cells, and bacteria (i.e., “pus”) in the pleural cavity [4]. The risk factors for empyema include pneumonia, chronic lung disease, diabetes mellitus, prolonged corticosteroid use, illicit drug use, alcohol abuse, aspiration, thoracic or esophageal surgery, or trauma [5]. Empyema has rarely been associated with hepatic abscess, with very few cases reported in the literature [2]. The implicated pathogens in community-acquired empyema are most commonly Gram-positive bacteria, especially the viridans group streptococci. Staphylococcus aureus, particularly methicillin-resistant Staphylococcus aureus and Pseudomonas aeruginosa, predominate in hospital-acquired cases [4,5]. Studies have shown that some of the organisms implicated in hepatic abscesses are Escherichia coli and Streptococcus spp [6,7]. Anginosus group streptococci are a member of the viridans group and are part of normal human flora. They are often implicated in disseminated pyogenic infection, which affects two or more of the following organs: liver, lung, spleen, and central nervous system [8,9]. A study found the presence of underlying dental infections, malignancy, gastrointestinal and respiratory tract disease to be associated with about 42% of cases of disseminated anginosus-group streptococcal infections [8]. Respiratory infections caused by this group are mostly seen in male patients with comorbid conditions. Anginosus-group streptococcal pneumonia can be complicated by pleural effusion and empyema in those with pleural effusion [10].

The mortality rate of empyema could be as high as 47% in hospital-acquired and 17% in community-acquired cases, while the mortality of hepatic abscesses can be as high as 95% if left untreated or managed nonsurgically [11,12,13]. The management of empyema usually involves the drainage of the fluid with tube thoracostomy and the administration of adjunctive antimicrobial medications. Although intrapleural fibrinolytic and mucolytic agents are sometimes available as options, they are not considered first- or second-line to tube thoracostomy [4,5]. The management of hepatic abscess includes both antibiotics and percutaneous drainage [13]. Most patients with hepatic abscess required prolonged percutaneous drainage with a median duration of 10 days [14].

In patients with concurrent empyema and hepatic abscess, hepatic abscess drainage is usually required after drainage of the pleura [1,2,3]. Remarkably, our patient had significant improvement in the hepatic collection after drainage of the empyema. The empyema in patients with combined empyema and a hepatic abscess is usually attributed to transdiaphragmatic extension of the hepatic abscess to the pleural space and development of a hepato-pleural fistula [2,15]. Due to the rapid resolution of the hepatic collection in our patient after the drainage of the empyema, it is possible that the hepatic abscess was due to the transdiaphragmatic extension of the empyema to the peritoneal cavity. There are no other reported cases of the resolution of hepatic abscess after the drainage of empyema in patients presenting with both empyema and hepatic abscess, making our case the first in the literature.

## 4. Conclusions

Concurrent empyema and liver abscess, although rare, should be a consideration in patients presenting with respiratory symptoms, fever, right-sided pleural effusion, and elevated liver enzymes. The prompt initiation of antibiotics and surgical drainage of the empyema is required to decrease mortality. Although not previously reported, the drainage of empyema can lead to the resolution of the hepatic abscess, which may suggest the transdiaphragmatic extension of the empyema into the liver rather than the transdiaphragmatic extension of the hepatic abscess into the lung leading to empyema. As such, drainage of the pleural effusion may be considered first in patients with concurrent empyema and liver abscess.

## Data Availability

Not applicable.

## Abbreviations

WBC (white blood cell), ALT (alanine transaminase), AST (aspartate transaminase), ECG (electrocardiogram), CXR (chest X-ray), INR (international normalized ratio), CT (computerized tomography).
